# Discovering Functional Modules across Diverse Maize Transcriptomes Using COB, the Co-Expression Browser

**DOI:** 10.1371/journal.pone.0099193

**Published:** 2014-06-12

**Authors:** Robert J. Schaefer, Roman Briskine, Nathan M. Springer, Chad L. Myers

**Affiliations:** 1 Biomedical Informatics and Computational Biology Graduate Program, University of Minnesota Rochester, Rochester, Minnesota, United States of America; 2 Department of Computer Science and Engineering, University of Minnesota, Minneapolis, Minnesota, United States of America; 3 Microbial and Plant Genomics Institute, Department of Plant Biology, University of Minnesota, Saint Paul, Minnesota, United States of America; Leibniz-Institute for Vegetable and Ornamental Plants, Germany

## Abstract

Tools that provide improved ability to relate genotype to phenotype have the potential to accelerate breeding for desired traits and to improve our understanding of the molecular variants that underlie phenotypes. The availability of large-scale gene expression profiles in maize provides an opportunity to advance our understanding of complex traits in this agronomically important species. We built co-expression networks based on genome-wide expression data from a variety of maize accessions as well as an atlas of different tissues and developmental stages. We demonstrate that these networks reveal clusters of genes that are enriched for known biological function and contain extensive structure which has yet to be characterized. Furthermore, we found that co-expression networks derived from developmental or tissue atlases as compared to expression variation across diverse accessions capture unique functions. To provide convenient access to these networks, we developed a public, web-based **Co**-expression **B**rowser (**COB**), which enables interactive queries of the genome-wide networks. We illustrate the utility of this system through two specific use cases: one in which gene-centric queries are used to provide functional context for previously characterized metabolic pathways, and a second where lists of genes produced by mapping studies are further resolved and validated using co-expression networks.

## Introduction

Despite our ability to rapidly sequence genomes, our understanding of gene function is still quite limited in most species. This lack of knowledge fundamentally limits our potential to understand biological systems and is particularly problematic in identifying causal genes for traits of interest. To address this challenge, one successful strategy has been the systematic integration of several types of genomic data. Interaction networks based on protein-protein interaction, localization, sequence, and expression have extensively been used in yeast, arabidopsis, and other model organisms to capture functional information, significantly improving prediction of gene function and characterization of interactions among gene products [1]–[4]. While many of these data types are not yet available in maize, there are numerous whole genome expression profiles available. Studies in model organisms show that simple gene expression analyses for diverse samples can provide a robust source of information when inferring functional relationships among genes [5], [6]. Co-expression, or correlation of gene expression among samples, can uncover genes that are co-regulated within a pathway or constrained to a specific tissue or sample [7], [8]. These relationships are captured by measuring co-variation for each pair of genes, which can then be intuitively represented as a network [7], where each node depicts a gene and each edge shows the magnitude of co-expression between them [9].

Co-expression-based approaches have already been used successfully to infer functional relationships in a variety of agronomically important organisms including rice, barley, tomato, and maize [8]–[13]. Specifically in maize, co-expression networks have helped to identify modules rewired between maize and its wild ancestor, teosinte, suggesting that the altered phenotypes of domestication are due to changes in regulation of expression [12]. Other work in maize has used co-expression to characterize developmental stages from embryogenesis to senescence [13] as well as to assess conservation of functional modules between maize and rice [14]. Likewise, co-expression relationships have been leveraged in tomato to discover novel candidate genes involved in lycopene and flavonoid biosynthesis metabolic pathways [9]. In rice, co-expression has been used to characterize genes related to drought stress and cellulose biogenesis [11]. In addition to helping characterize the function of unknown genes, network-based methods have also been widely used to prioritize sets of candidate genes in relation to a trait of interest, changes across different tissues, conditions, or genotypes [9], [15].

While co-expression analysis is a powerful approach, these networks are quite large, difficult to explore, and are cumbersome to share or recreate [16], [17]. Even when a stringent threshold is applied to the edges in a co-expression network, basic network visualization and gene extraction becomes difficult when the networks grow beyond a few thousand nodes. In order to address these challenges and add value to existing maize gene expression data, we built, characterized, and contrasted two co-expression networks generated from whole genome expression profiles of maize. We first demonstrate the validity of these co-expression networks by examining both unique and common enrichments for known biological functions in both co-expression networks as well as the extensive amount of yet uncharacterized structure present in both networks. Additionally, to facilitate convenient analysis of these networks, we introduce a comprehensive web resource called **COB**, the **Co**-expression **B**rowser, which allows users with a set of genes of interest to explore the same co-expression networks used in our analysis over the web. We illustrate the features of COB through two specific use cases, including recovery of well characterized metabolic and developmental pathways as well as the identification of candidate genes that fall within previously described quantitative trait loci.

We note that the primary focus of this manuscript is to demonstrate the utility of these maize co-expression networks and illustrate some of the common uses of the system, for which we provide a relatively comprehensive discussion. We have chosen not to emphasize technical details of the implementation of the COB system here for the purpose of clarity. However, we do describe the key aspects of our implementation (see Materials and methods). The source code for the interface and complete database are freely available under the MIT license and can be downloaded by visiting: http://csbio.cs.umn.edu/cob.

## Results and Discussion

### Construction of co-expression networks

Two distinct transcriptome datasets were used to build separate gene co-expression networks [12], [18]. The first network was built using expression profiles from a single tissue (8-day seedlings) from 62 genotypes that included diverse maize and teosinte samples. This ‘*genotype’* network was generated using expression data for 18,242 high confidence genes (4a.53, www.maizesequence.org) which were pre-filtered based on comparative genomic hybridization (CGH) data in order to minimize differences due to sequence variation from the reference genome [12]. The second co-expression network was constructed using gene expression data from 60 different tissues/stages of a single reference genotype, B73, constituting a *'developmental'* network that characterizes the variation of gene expression patterns among different tissues within the same genotype. The developmental co-expression network was generated from a set of 23,331 high confidence genes mapped to the 4.53a filtered set [19] that were expressed in at least one tissue [18].

While additional, publicly available, gene expression profiles exist for maize, the use of two large datasets derived from two individual experiments provided a balanced sampling of tissues and genotypes and reduced the need for complex normalization to account for cross-platform or cross-lab systematic variation. Furthermore, studies in yeast and arabidopsis found that datasets consisting of ∼50–100 profiles provided sufficient variation for constructing co-expression networks [7].

Co-expression network interaction scores were calculated using the Pearson correlation coefficient, which was then Fisher-transformed and normalized to a standard normal distribution using the standard score statistic (Z-score hereafter) (Figure S1). This transformation guarantees that the sampling distribution of a correlation coefficient derived from a bivariate normal distribution will be normal with a defined variance that relates to the size of the vectors being correlated [20]. Applying this transformation enables direct comparison of correlation values across networks, even when they are derived from a different number of samples [5]. At a relatively stringent co-expression threshold (Z>3), 16,440 developmental network genes (70%) and 15,506 genotype network genes (85%) had at least one significant interaction. A total of ∼1.2 million and 598,000 significant interactions were discovered in the developmental and genotype networks, respectively, using the gene sets described above. The degree distributions of both networks were fit best by a truncated power law distribution (Figure S4; See materials and methods), which is consistent with co-expression networks in other species [21]. Direct comparison of significant interactions between the networks is complicated by the fact that partially distinct sets of genes were included in each dataset (Figure S2). A set of 13,514 common genes are expressed in both the genotype and development expression profiles and 8,842 (65%) of these genes have significant interactions in both networks (retained common, RC, genes hereafter). Of these RC genes, there are substantially more significant developmental interactions (554,707) than genotype interactions (177,392). In the case of both all genes and of RC genes, 6,980 interactions were significant in both networks. The number of conserved interactions (6,980) between the networks was significantly higher than expected by chance (P<2e-10; hypergeometric test) showing that both networks capture non-random relationships between a core set of retained common genes.

### Co-expression networks are enriched for biological function and show unique functional characteristics

**Figure 1:**
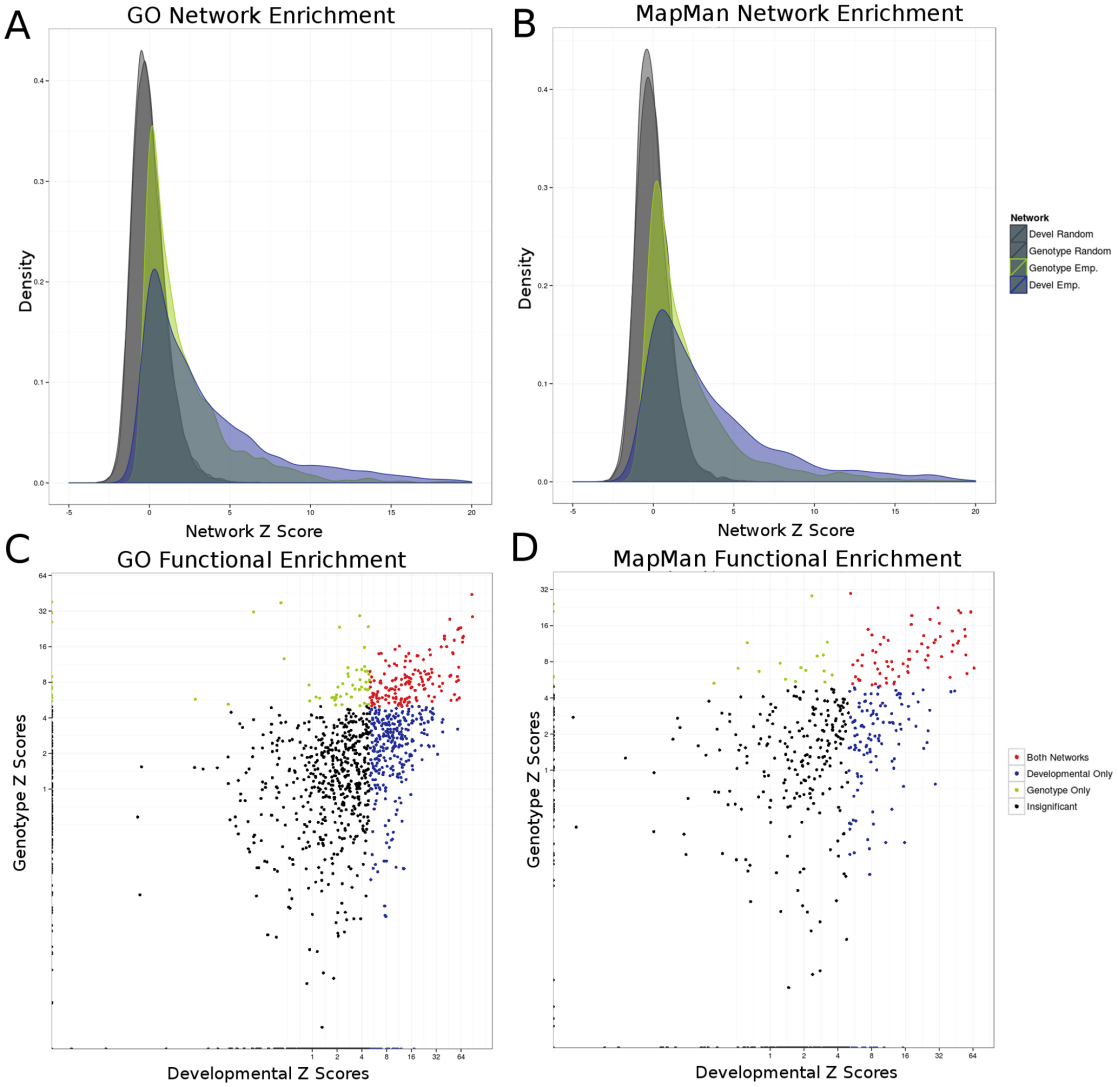
Enrichment for GO and MapMan terms in co-expression networks. Enrichment of highly co-expressed interactions among sets of genes in the network was calculated using both the Gene Ontology (GO) standard as well as the MapMan (MM) standard using the Z-test. Interactions among random sets of genes with the same size as GO and MM were calculated and compared to empirical interactions showing that interaction densities in empirical data are stronger in both networks for both GO (**A**) and MM (**B**). Individual annotation terms are plotted against each other in each network (**C–D**) showing that certain terms are more represented in a single network (green and blue points) or represented by both networks (red points).

One common approach for assessing biological information within co-expression networks is to test for enrichment of curated functional annotations [8], [22]. The enrichment for co-expression among genes with similar functional annotations from the Gene Ontology [23] or MapMan [24] was tested relative to the null expectation that each gene set should exhibit no difference from background in the average co-expression level [25] using the Z-test (Figure 1 A–B). After Bonferroni correction for multiple hypothesis testing (p_adj_<0.05), many of the GO (796/3318; p_raw_<1×10^−5^) and MapMan (217/1957; p_raw_<2×10^−5^) groups had significant (Z>5; p_adj_<0.05) enrichments for interactions, indicating that these co-expression networks are capturing coherent information (Figure 1 C–D). A subset of these gene sets (GO: 467 Developmental, 114 Genotype; and MapMan: 126 Developmental, 22 Genotype) were only effectively captured in one network (Figure 1 C–D). The developmental network was uniquely enriched for metabolic and cellular transport (GO:0071702), cell wall synthesis (GO:0070882) and aerobic respiration (GO:0009060) in addition to many others (Table S1 in File S1). Similarly, enriched MapMan groups for the developmental network included the chloroplast (29.2.1.1.1.1.4), light reaction (1.1.5.3), and metabolism (25.1) (Table S1 in File S1). The genotype network was uniquely enriched for GO terms relating to the electron transport chain (GO:0022900), DNA repair (GO:0006298), and various regulatory processes (GO:0051493, GO:0032271, GO:0030833, GO:0008064, GO:0032956, GO:0043254, GO:0044087, GO:0032970, GO:0030832; Table S1 in File S1). MapMan terms uniquely enriched in the genotype network included annotations related to lignin biosynthesis (16.2.1.1010), electron oxidation/reduction (1.1.5), and ‘amino acid metabolism.degradation.histidine’ (13.2.7) (Table S1 in File S1).

In addition to unique functional enrichment in the two networks, there were many gene sets (215 GO; 69 MapMan) that were significantly co-expressed in both networks. Enriched MapMan terms in common included DNA synthesis (28.1.1), ribosomal related processes (29.2.1), and protein folding (29.6). GO terms significantly enriched in both networks included photosynthesis (GO:0015979), microtubule based movement (GO:0007018), DNA replication (GO:0006260), and chromatin assembly (GO:0031497) (Table S1 in File S1). In both GO and MapMan gene sets, the developmental network captured more unique annotations than the genotype network. Together, these findings suggest that the both networks effectively capture many already characterized biological processes, indicating that the networks contain biologically relevant information. However, the networks are complementary in what they capture and many specific processes are better captured by a single network, which could be an important factor in choosing a network for analysis related to a specific biological question.

### Network clusters provide a functional context

While gene set enrichment can bolster confidence that network interactions contain biologically relevant information, it fails to uncover structure that was previously unknown. Unsupervised approaches such as network clustering allow novel information to be revealed by examining structure that is present in the co-expression networks.

**Figure 2:**
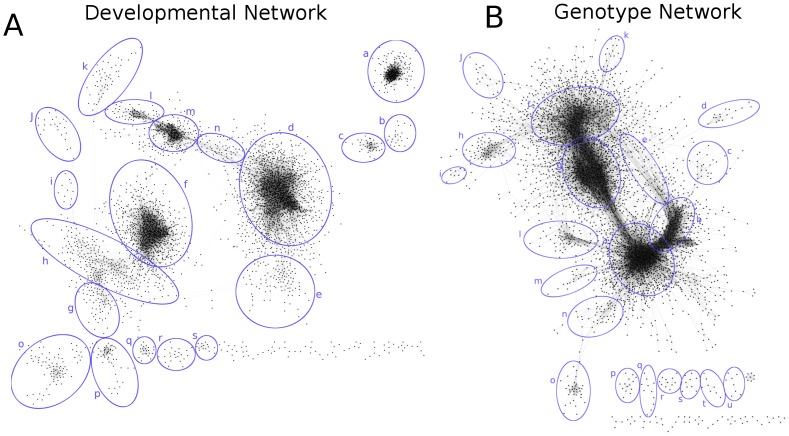
Clustering of co-expression networks. The strongest 100,000 interactions in each network were extracted and node positions were calculated using a force-directed algorithm which simulates interactions as springs while iteratively pushing nodes outwards. Highly connected nodes form natural clusters (circled in blue) in two dimensional space and are grouped based on their connectedness which is easily interpreted visually. The graph clustering algorithm, MCL, was also applied to the interactions. The resulting clusters overlapped with the global-scale clusters from the force-directed layout (Tables S3 and S4 in File S1).

Prominent topological features were extracted from the network by taking the strongest 100,000 edges to generate a two-dimensional layout for each network using a force-directed algorithm (Figure 2) [26]. We selected 19 distinct developmental clusters and 21 genotype clusters to examine for functional coherency in an attempt to provide some biological context for the high-level structure that is visually apparent (Figure 2; genes within each cluster can be found in Table S2 in File S1). We also defined clusters more systematically by applying a graph-based clustering algorithm, MCL [27], which showed agreement with those identified visually, though often separated the large visually defined clusters into smaller sub-clusters (Tables S3 and S4 in File S1). Several of the clusters derived from each network contained functionally coherent sets of genes: 13 of 21 developmental clusters and 11of 19 genotype clusters showed significant enrichment for GO process terms (nominal p-value<0.05). Even in clusters with enrichment, a large number of genes in each cluster had no GO annotations. For example, developmental cluster A had a total of 184 genes but across all GO processes that were enriched, an average of 17 and a maximum of 36 genes were annotated to a process, leaving the majority of genes in the cluster uncharacterized. Cases where a set of functionally coherent genes clusters with a large set of uncharacterized genes are worthy of further study and are a key strength of co-expression network analysis. A full compendium of Gene Ontology and MapMan enrichment for all clusters can be found on our accompanying website (http://csbio.cs.umn.edu/cob/paper).

**Figure 3:**
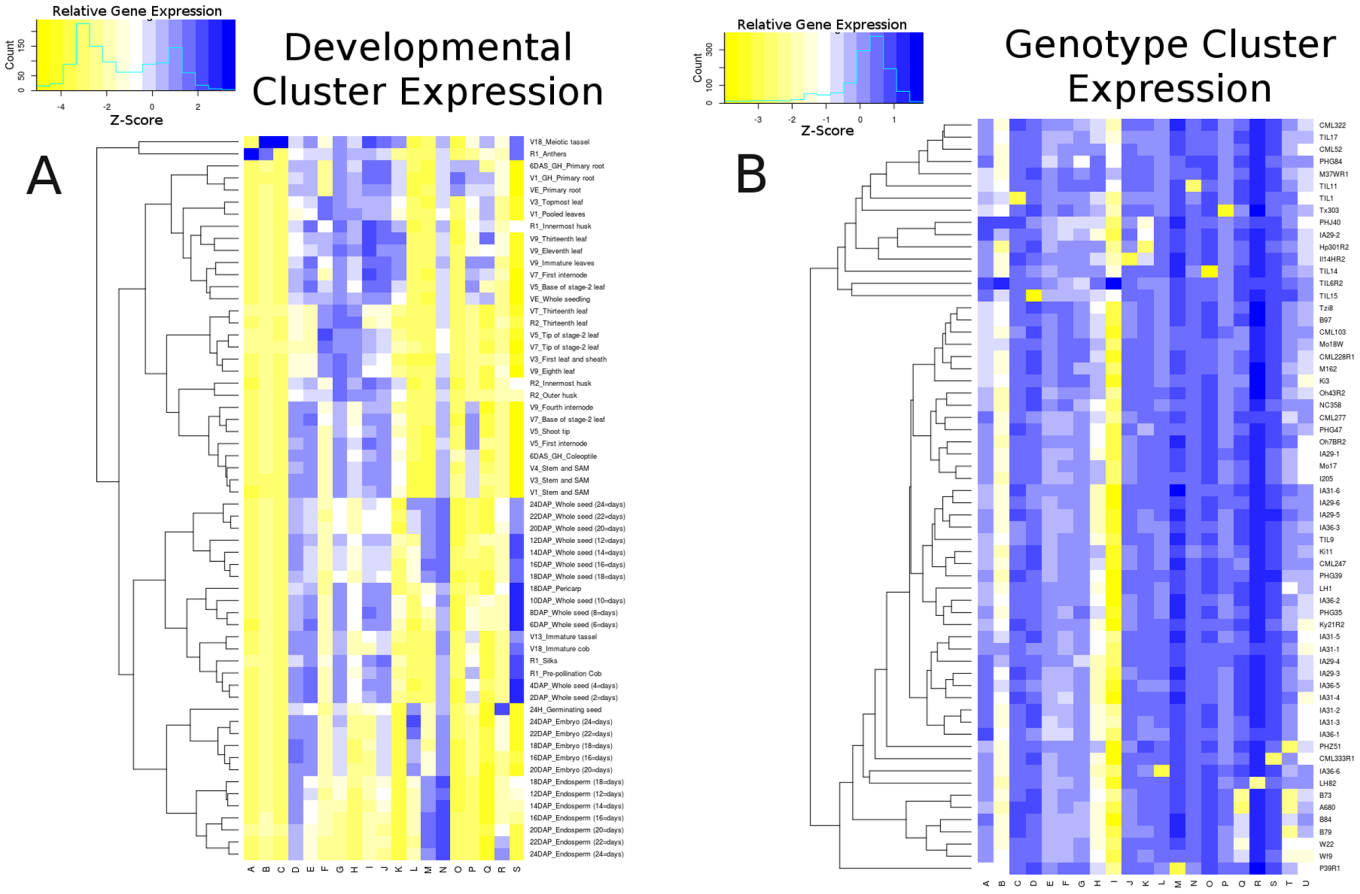
Expression patterns driving co-expression network clusters. Clusters identified in Figure 2 (shown on horizontal axis) were further broken down into raw expression components by sample (shown on vertical axis). Expression patterns for each cluster of highly co-expressed genes are different for each cluster. Raw expression values are normalized compared to global background expression levels of genes within a cluster indicated here with the color white. Blue indicates over expression while yellow indicates under expression of a gene cluster. (**A**) shows developmental clusters while (**B**) shows genotype clusters.

**Figure 4:**
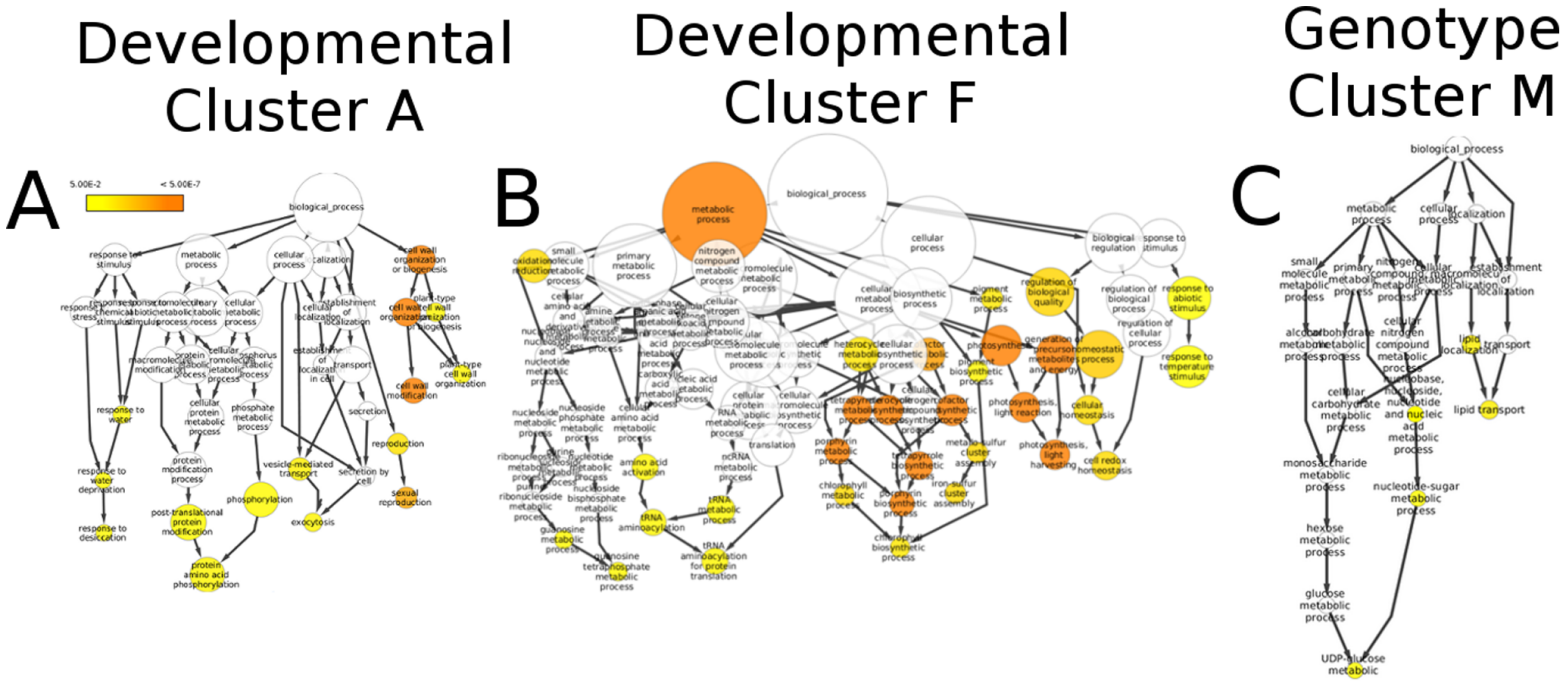
GO enrichment analysis of co-expression clusters. Gene clusters identified in Figure 2 were examined for enrichment of Gene Ontology terms. (**A**) Developmental cluster A, which exhibited a strong signal for expression in the anthers (see Figure 3), is enriched for GO terms related to sexual reproduction, response to desiccation, and cell wall biogenesis/modification. (**B**) Developmental cluster F, highlighted by patterns of expression in the leaves, is notably enriched for terms annotated for photosynthesis, response to temperature stimulus, and chlorophyll metabolism. (**C**) Genotype cluster M exhibits drastic under-expression in the P39 genotype, a sweet corn line, and shows significant GO enrichment in terms related to UDP-glucose as well as nucleotide-sugar metabolism and lipid transport.

An examination of the expression patterns for each cluster of co-expressed genes reveals striking differences in the trends that lead to clusters in the two networks (Figure 3; See Materials and Methods). Patterns of co-expression observed within the developmental network are largely the result of high expression levels in a small number of tissues. For example, developmental cluster A is driven distinctly by expression in the anthers. When examined for functional enrichment, cluster A was enriched for genes related to sexual reproduction (GO:0019953), cell wall modification (GO:0071555), and response to desiccation (GO:009269) (Table S5 in File S1). These functions are consistent with biological processes that occur in the anthers, suggesting that cluster A is effectively capturing relationships among genes specific to anther function (Figure 4A). Developmental cluster F, which was mainly driven by expression in the leaves, was enriched for processes related to photosynthesis (GO:0015979), oxidation-reduction (GO:0055114), and temperature response (GO:0009266) again recovering coherent, biologically relevant functional information (Figure 4B; Table S5 in File S1). Other clusters exhibited a similar pattern of high expression in a small number of samples though lacked enrichment for functional annotations. For example, developmental cluster B has a very similar expression pattern compared to cluster A, having high expression in the anthers. A lack of significant enrichment could indicate that there are additional biological relationships that remain to be characterized.

In contrast to the pattern of high expression in one or a small subset of samples observed in the developmental network, approximately half of the clusters in the genotype network often resulted from significantly lower expression in a single or small number of genotypes (Figure 3B). Genotype cluster M, which is characterized by strong under-expression in maize line P39, was enriched for ontology terms annotated for carbohydrate metabolism (GO:0005975) and UDP-Glucose metabolic processes (GO:0006011) (Figure 4C). This pathway is involved in glycosyltransferase reactions that play a role in the biosynthesis of saccharides. Interestingly, P39 is a sweet corn line that was selected for starch synthesis properties in the kernel tissue. Finding genes involved in starch metabolism exhibiting altered expression patterns in vegetative tissues suggests that some of the variation in this sweet corn line possibly affects tissues beyond the kernel.

One possibility that may explain this pattern of low expression in a single genotype is that genetic variation, not expression variation, results in less efficient probe hybridization. To explore this possibility, we tested each cluster for enrichments of genes with known genetic variation as determined through array CGH on the same lines [28] (Table S6 in File S1). Out of 21 clusters in the genotype network, only two (clusters B and D) had significant enrichment for genes for which variation was detected as well as concordance between decreased expression and decreased genomic DNA hybridization in the same line (Table S6 in File S1, see Materials and Methods for details). Even in these cases, the number of genes with evidence for genomic variation comprised a small minority of the total cluster (15 of 375 for cluster B, and 3 of 17 for D). Thus, we conclude that genetic variation is not a major driver of this pattern in the genotype network.

### COB — the co-expression browser

To enable broad access to the co-expression networks, we built a comprehensive web resource called the **CO**-expression **B**rowser, or COB, which can be accessed at http://csbio.cs.umn.edu/cob. COB was designed around a few key design principles and paradigms, which are briefly discussed below.

#### Simple query system

Upon a user's initial visit to the site, there is a simple query box that can be used to query a single gene (either maize classic names or Gramene build 4.53a gene ids e.g. GRMZM2GXXXXXX), a short list of genes or a set of genomic coordinates. For example, querying for *adh1* in the developmental dataset results in 512 highly co-expressed genes. Due to rendering constraints, a maximum of the 65 strongest co-expressed genes are drawn in the network view; however, a complete dataset is returned and displayed in table format in the table panel. This large network can either be manually thresholded by restricting the neighborhood size or interesting genes can be isolated in the gene list and can be re-queried simply by highlighting relevant genes and clicking the “ReQuery Selected” button. Un-rendered genes from the data pane can be added to the network by clicking them. This iterative process allows for larger networks to be thresholded down to manageable sizes, and conversely, allows small networks to be grown by iteratively adding additional genes, without the need to enter any visualization parameters upfront.

#### Integrated Tables and Networks

Once a network is ready to be displayed, two separate information panels are loaded. A graphical view of the network is shown along with a tabbed panel displaying various options and tables. Networks contain two useful pieces of information: (1) the set of genes included in the network, which is better viewed in a table, and (2) the set of gene interactions which are better suited to be displayed graphically. By integrating information displayed in each panel, interesting genes from the table can be quickly found in the network by clicking on the gene name in the table.

#### Network Level Exploration

Representing relationships as networks offers the benefit of easily overlaying additional information. Subsequent bioinformatic investigation of networks is a crucial step in narrowing down potentially interesting relationships based on a query gene. Relationships among genes in a network often need to be interpreted manually, although integrated tools make assessing putative associations much easier [22]. COB implements several basic bioinformatics network tools which allows for exploration based on the currently loaded network as well as the ability to download any loaded network for additional analysis. The information panel includes a tab called “Explore Network” which contains several basic bioinformatic features such as changing gene labels and dynamically loading which co-expression dataset interactions are shown. Basic functional analyses can be performed, including GO enrichment or highlighting genes from a specific locus of interest. Bulk coordinate information can be imported using the Import Tool, which allows for quick identification of genes on a specific chromosome or within a quantitative trait locus (QTL). Obtaining GO enrichment is possible for either the entire loaded network or for a subset of highlighted genes. Each significantly enriched GO term shows a short description, a hyperlink to its full description, and an option to highlight the genes in the network which are present in the ontology term.

#### Gene Level Exploration

While networks are efficient at displaying relationships among genes, they fail to efficiently show detailed gene information such as chromosomal location, alternative names, or orthology. In COB, gene details are interactively loaded from the server and include annotations such as alternate gene names, locus information, neighboring genes, available Gene Ontology, arabidopsis ortholog information, and links to other databases (See Materials and Methods).

### COB: Example Use Cases

The features of COB outlined above aim to provide simple access to information within co-expression networks, but are also designed around specific use cases. In order to demonstrate how COB might be useful to a researcher wishing to learn more about interactions among their genes of interest, we describe two use cases. The first examines whether a specific biological function, such as a biological pathway, is well captured by the co-expression network and investigates which genes are putatively associated with the pathway. The second illustrates how our co-expression resource can be used to pinpoint interesting candidate genes within a QTL region.

#### COB use case I: Recovering metabolic pathways

**Figure 5:**
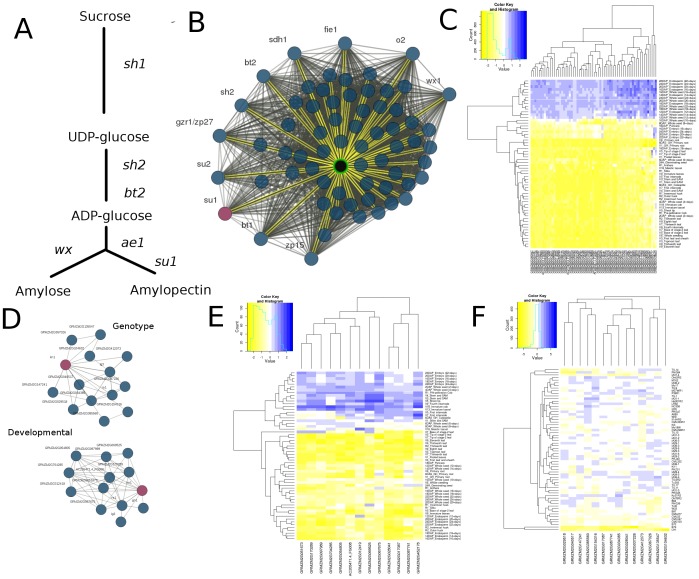
COB Use cases. COB was queried for *su1* which is known to be involved with the starch synthesis and sugar metabolism pathway (**A**). (**B**) shows the 66 highest co-expressed genes with *su1* including many genes already known to be involved with starch synthesis. Examining tissue expression patterns (**C**) shows that genes in the network are over expressed in whole seed and endosperm. (**D**) *KN1* was queried in both networks and recovered other known homeobox genes (*lg3, rs1, gn1*). (**E**) Patterns of gene expression show that these networks are driven by expression in varying stages of embryo development as well as the SAM and shoot tip. (**F**) Patterns of expression among genes within the *KN1* network among diverse maize genotypes.

Several classical maize genes or pathways were selected to demonstrate potential use cases for COB. The starch synthesis pathway is relatively well-characterized in maize [29]. A simplified version of the pathway including some of the best characterized maize genes is shown in figure 5A [30]. The *sugary1* (*su1*) gene was used to query COB using the developmental co-expression network (Red node; Figure 5B). Visible labels in the network were changed to ‘Common Only’ and the network layout was changed to ‘Force Directed’ in the ‘Explore Network’ tab. Patterns of gene expression among tissues show that relationships in the network are driven by over-expression in whole seed and endosperm and under-expression in vegetative, root and embryo (Figure 5C). A large group of co-expressed genes is identified and this network includes a number of the other maize genes that are known to play roles in starch synthesis or metabolism including *su2*, *bt1*, *bt2*, *sdh1*, *o2*, *sh2* and *wx1* (Table S7 in File S1) [31], [32]. In addition to containing a number of genes known to play roles in starch metabolism there are also other genes in this network with putative annotations that suggest they may play a role in starch metabolism including GRMZM2G024131 (Green highlighted node; Figure 5B) whose best arabidopsis ortholog is annotated to be involved in UDP-glucosyltransferase (Table S7 in File S1). Using tools available in COB, GRMZM2G024131 was located and selected from the ‘Gene List’ table in the ‘Main’ panel, highlighting the gene in the network. Neighboring interactions can be highlighted in yellow by clicking the ‘Neighbors’ header in the ‘Explore Genes’ tab, which reveals all direct edges including several previously characterized starch genes. This use case illustrates the ability to recover multiple genes in the same pathway while simultaneously uncovering additional, previously uncharacterized genes that may have related function.

One of the best characterized developmental mutants in maize is *kn1*. Dominant mutant alleles of k*n1* result in leaf developmental abnormalities [33]. The *kn1* gene encodes a homeobox protein and is normally expressed in the shoot apical meristem and involved in determination of meristem identity along with other related homeobox genes [34]. The *kn1* gene was used to query both the developmental and genotype (Figure 5D) co-expression networks. The developmental co-expression network of *kn1* includes other homeobox genes that have related functions such as *lg3*, *rs1*, and *gn1* (Figure 5D; Table S8 in File S1) [35], [36]. In addition, nine other genes also exhibit similar co-expression patterns which are driven by over-expression in developing tissue including embryo, shoot tips and shoot apical meristem (Figure 5E). These include several putative transcriptional regulators (Table S8 in File S1). The genotype co-expression network of *kn1* includes *lg3* and *rs1* and 11 additional genes (Figure 5D; Table S8 in File S1). Recent ChIP-seq experiments have identified the binding sites of *KN1* in the maize genome and uncovered many genes that are putatively regulated by *KN1* [34]. We assessed whether the co-expressed genes were direct targets of *KN1* regulation. While 10.8% of all maize genes have a KN1 binding site within 10 kb, 66% of the developmentally co-expressed genes and 31% of the genotype co-expressed genes have *KN1* binding sites nearby (Table S8 in File S1), providing evidence that this sub-network captures a set of coherently regulated genes.

While the two above use cases show how COB might be used to characterize already known pathways, we anticipate that this guilt by association approach would also be useful in examining putative function of uncharacterized candidate genes and pathways. In situations where little is known about a target, examining relationships is useful for integrating independent sources of information as demonstrated by our use case of *KN1* regulation targets.

#### COB use case II: Augmented QTL candidate gene discovery

Co-expression networks may also provide an opportunity to improve the identification of candidate genes underlying quantitative trait loci (QTL) by examining co-expression with “bait” genes which are known to be related to specific phenotypes with genes that are located in genomic regions corresponding to trait QTL. We investigated the potential to use COB to integrate co-expression data with QTL mapping information in order to identify candidate genes in QTLs previously linked to leaf angle [37].

As reported by Tian *et al.*, 30 QTL regions containing over 3,700 possible causal genes have been shown to be linked to leaf angle [37]. Given this large list of plausible genes, co-expression analysis provides a means to further resolve causal candidate genes which lie within these loci. Classic maize genes liguleless1 (*lg1*) and liguleless2 (*lg2*) are located in the two most significant QTL regions identified by the study and have significant genome-wide association study (GWAS) markers associated with leaf angle [37]. While genes *lg3* and *lg4* were not present in QTL linked to leaf angle, they are known to have the potential to affect this trait and thus were included as bait genes [35]. Querying COB using all 4 liguleless genes as bait, we used the locus filtering tool to extract co-expressed genes within one of the 30 QTL identified by Tian *et al.*, and identified three candidate genes in the genotype network as well as a single candidate gene in the developmental network which were within QTL regions as well as significantly co-expressed with at least one *lg* ‘bait’ gene.

From the developmental network, we extracted GRMZM2G110834 which is co-expressed with *lg4* among 13 other genes in the developmental network. This gene is related to arabidopsis gene AT1G15110 which encodes a putative phosphotidyl serine synthase family protein. It is located on chromosome 3 (26,091,050) and has three significant SNPs associated with leaf angle within 150KB of this gene (See Materials and Methods).

Likewise from the genotype network, we extracted GRMZM2G054621 which is highly connected in the genotype network (>70 interactions) and was found due to its co-expression with *lg4*. In this network, there are several other strongly co-expressed genes including a direct interaction with classic maize gene *rs1*, which is known to affect ligule development [38]. There are ten significant GWAS hits located within 1Mb of GRMZM2G054621, including one significant marker within 150kb having no intervening genes. GRMZM2G054621 is also highly connected in the development network (>700 edges), including connections with *lg4*.

GRMZM2G037226 is well connected to a small genotype network which includes several genes known to affect leaf development or formation (*lg3, kn1, rs1*) [35]. This gene encodes a protein with RNA-binding domains and its arabidopsis ortholog (AT2G41060) has interesting functional annotations related to leaf senescence and ethylene biosynthetic processes and localization [39], [40]. GRMZM2G037226 is located on chromosome 10 (144,024,471) and has three moderately significant GWAS hits within 200 kb of this gene though there are several intervening genes.

Using multiple sources of independent data, the above use cases demonstrate how COB can be used to further investigate lists of candidate genes commonly generated using traditional genetic mapping approaches. The high accuracy, but low resolution offered by mapping techniques can be supplemented with further functional analysis. Of the over 3,700 candidate genes that lie within leaf angle QTL, only a handful were significantly co-expressed with genes previously identified to affect ligule development demonstrating the utility of COB in efficiently filtering lists of candidate genes as well as integrating other functional information. Further analysis of these candidates is required to confirm their causal link to leaf angle, but co-expression network analysis can play a key role in the process.

## Conclusions

In this study, we examined two genome-wide maize transcript expression datasets and characterized their functional properties. We found that the networks are highly enriched for characterized functional relationships, suggesting they will be useful as a resource for understanding gene function in maize.

Examining two different networks (genotype versus developmental) revealed that each network recovers unique functional enrichments. These relationships, especially in the case of gene co-expression, are sensitive to context, and different collections of expression profiles can dramatically influence the functional information contained within a co-expression network. In addition, we examined which accessions/tissues were strongly influencing co-expression and found patterns of co-expression that differed between clusters. In an age of widely available expression data sets, it becomes increasingly important to consider the context in which the data were derived.

Although co-expression cannot decisively assign function to genes, it does provide a means to further examine meaningful relationships between genes. Even with extremely powerful methods such as QTL mapping or genome-wide association analysis, candidate regions still contain possibly thousands of candidate genes. Using co-expression networks to reduce or rank candidate genes can be a robust approach for examining genes responsible for complex traits in maize. With COB, our web-based platform, this type of analysis can be readily performed using the same data and tools described in our specific use cases.

It is inevitable that a wealth of additional data will be generated by various other gene expression projects and newly available sequencing information generated by the next generation of maize mapping populations. Networks are natural, interpretable structures that allow relationships to be explored in an intuitive way. Not only are we interested in further extending the functionality and scope of COB, including additional co-expression networks and toolsets as these data become available, we invite anyone interested to contribute to our code base. COB is freely available software under the MIT license and hosted publicly in a repository which can be accessed by directly visiting COB.

## Materials and Methods

### Raw Expression Data and Co-expression Networks

Raw expression values were obtained directly from sources mentioned in Swanson-Wagner *et al*. [41] and Sekhon *et al*. [42]. Briefly, custom NimbleGen arrays captured expression profile for 18,242 CGH filtered genes which were aligned to the B73 reference genome. Expression matrices were used to generate profile correlations for both the developmental networks and genotype networks by calculating the Pearson correlation coefficient between each pair of gene expression profiles in each instance. Calculations were performed using the Sleipnir C++ library [43]. To facilitate comparisons across networks, we applied the Fisher Z transformation to both correlation matrices. Z-transformed values were then normalized by dividing by the standard deviation in order to obtain an approximately standard normal distribution, making scores comparable across networks.

### Analysis of Enrichment for Genetic Variation Underlying Genotype Network Clusters

Because genetic variation across the diverse set of maize lines could possibly result in the appearance of co-expression in the genotype network, we analyzed the connection between clustering in this network and genetic variation. We used data derived from comparative genomic hybridization (CGH) on the same set of lines use to build the genotype network [28]. First, any probe with low CGH signal in 3 or more lines was filtered from consideration in the expression analysis, which resulted in a total of 46,167 remaining probes measuring expression in 18,242 genes (1–4 probes per gene) for co-expression analysis. Using the clusters described in this manuscript, we further assessed if any clusters were enriched for genes showing copy number or presence/absence variation (CNV) in the CGH data, which revealed that 3 of 21 genotype network clusters had enrichment for genes with CNV (p<0.05). The genes in these clusters were then analyzed for concordance between the pattern of CNV and the expression profile across the lines. Specifically, the expression values for genotypes that exhibited low CGH signal relative to the B73 reference in more than 50% of the CNV-associated genes in that cluster were evaluated for decreased expression. Each genotype's average expression across the genes in the cluster was compared with the average in other genotypes to compute a Z-score measuring the difference from the average genotype for this cluster as well as its rank relative to all other genotypes (low rank corresponds to low expression). 2 of the 21 clusters exhibited both enrichment for CNV genes and exhibited at least moderate concordance between the expression profile and CNV profile. Even in these cases, only a minority of genes within each cluster had a CNV. The results from these analyses are presented in Table S6 in File S1.

### Gene Ontology and MapMan Enrichment

Annotations for GO (release 4a.53) and MapMan (Zm_GENOME_RELEASE_09.txt) were downloaded from http://ftp.maizesequence.org/release-4a.53/functional_annotations/ and http://mapman.gabipd.org/web/guest/mapmanstore respectively. Using all possible interactions in each network, all pair-wise interactions between genes within the same annotation were compared to the network background which had a standard normal distribution using the Z test. Significance was assigned to terms based on Bonferroni corrected p-value of <0.05. Points were plotted as Genotype vs. Developmental (Figure 1) in order to visualize network specificity in each annotation for both GO and MapMan.

**Global Features and Cluster Discovery**

The top 100,000 edges in both networks were chosen based on rendering constraints (∼10–20% total significant edges) to extract the most prominent network features. These most significant edges were used in a spring-embedded layout algorithm implemented in the graph visualization program Cytoscape to generate a global network layout [26]. This algorithm provides an intuitive view of the global-scale features of the network. 19 developmental and 21 genotype clusters were chosen visually; gene lists can be found in Table S2 in File S1. The Markov Cluster Algorithm (MCL) was also used to generate sub-networks systematically [27]. MCL inflation parameters were run at level of 1.2, 1.4, 1.6, and 2. Visually striking clusters were compared to MCL clusters with inflation parameter of 1.4 which provided sub-networks similar in size to those chosen visually shown in Tables S3 and S4 in File S1.

### Cluster Functional Enrichment

Clusters derived above were examined for functional enrichment using both Gene Ontology (GO) and MapMan (MM). Enrichment was assessed under the null expectation that co-expression among genes which are non-functional would be at background levels. Co-expression was assessed between genes within gene sets and compared to background co-expression using the Z-test (as the correlation data were previously Z-transformed and normalized, the expected distribution is standard normal if there is no correlation structure present):
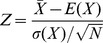



### Cluster Heat Maps

Raw gene expression data was extracted for each gene within a cluster. Log fold expression ratios for each gene were normalized by subtracting the gene averages then dividing by the standard deviation for each cluster. Yellow values show negative mean log fold deviation from cluster average and blue shows positive deviation. Hierarchical clustering was performed on the samples used in each network (tissues/time-points for developmental network and genotype for the genotype network) using the heatmap.2 package in R.

### COB: The Co-expression Browser

The COB system consists of an event-driven client side interface implemented in html/javascript and PHP coupled to a database-driven backend using JSON and AJAX. The underlying raw and thresholded co-expression values were stored in a MySQL database for easy access via COB (Figure S3). Tables were optimized to return co-expression results by indexing co-expression interactions using B-Trees as implemented within MySQL. The COB database and web portal are served from a Solaris based webserver running SunOS 5.10 with Dual Core Intel Xeon(R) 2.53 GHz CPUs and 80 G of RAM. Information is passed from the client to the server using AJAX and the network is represented graphically using the Cytoscape Web library [44]. The code is publicly available under the MIT software license and can be downloaded by directly visiting the website. COB is actively being developed in a separate experimental branch while a stable version can always be accessed at http://csbio.cs.umn.edu/cob.

### Network Degree Distribution Analysis

Degree for each gene was calculated at a significance cutoff of Z> = 3. Degree cumulative distribution functions were calculated and plotted using the python package 'powerlaw' [45]. To account for logarithmic binning of degree, best fit complementary CDF curves for power law, exponential, and truncated power law were plotted for each network. Candidate distributions were all compared using the “Fit.distribution_compare” function in the powerlaw package, which reports log likelihood ratios between the two candidate functions. Results from this analysis can be found in Figure S4.

### QTL candidate Discovery

Genetic coordinates for leaf angle SNPs and QTL were extracted from Table S3 from Tian *et al*. [37]. QTL support intervals were imputed to AGP positions based on known physical positions of flanking NAM markers. COB was queried for genes which lie within the QTL support regions each for leaf angle, length and width in addition to bait genes: lg1, lg2, lg3 and lg4. Genes co-expressed with bait genes were characterized based on their known ontological functions, proximity to known leaf architecture SNPs and known arabidopsis orthologs.

## Supporting Information

Figure S1 **Normalization of pairwise correlation values.** Histograms show pre- and post-normalized values for all pairwise interactions in genotype and developmental networks. Distributions are approximately normal, with the exception of heavy tails reflecting correlation structure among genes, and Z-score transformed distributions are comparable across networks.
(TIFF)

Figure S2 **Comparison of gene sets in genotype and developmental network.** Different starting sets of genes were used in the two experiments of which only 13,514 genes overlapped (**A**). Gene which retained at least a single significant co-expressed interaction are considered depending on whether the entire gene set was used (**C**) or only the union of the two data sets were considered (**B**). Similarly, corresponding edges were considered based on if they were calculated with all common genes (**D**), retained common genes (**E**), or simply all genes (**F**).
(TIFF)

Figure S3 **COB database schema.** COB Database schema showing relationships among datatypes used in COB. Tables were designed both for query speed as well as optimized for large insertions.
(TIFF)

Figure S4 **Network degree distributions and assessment of fit to candidate distributions.** Degree distributions were assessed in each network individually at an edge significance cutoff of Z> = 3. Best fit lines for each distribution were plotted with degree against the probability of observing a degree larger than X. Bins were logarithmically spaced in order to control for the heavy tail. Loglikelihood ratios are inset comparing fits of different heavy tailed distributions commonly observed in other networks. Positive ratios reflect a higher likelihood of the first listed distribution.
(TIFF)

File S1 **Tables S1-S8. Table S1.** Network GO and MapMan annotations. **Table S2**. Genes in clusters identified in Figure 2. **Table S3**. Overlap of developmental clusters identified by MCL (vertical axis) and clusters identified in Figure 2 (horizontal axis). **Table S4**. Overlap of genotype clusters identified by MCL (vertical axis) and global clusters identified in Figure 2 (horizontal axis). **Table S5**. Gene Ontology enrichments for developmental clusters A, F and genotype cluster M. **Table S6**. Concordance between clusters with known structural variation and gene expression. Genes in these clusters were analyzed for concordance between the pattern of CNV and the expression profile across all genotypes. Expression values for genotypes that exhibited low CGH signal relative to the B73 reference in more than 50% of the CNV-associated genes in that cluster were evaluated for decreased expression. Each genotype's average expression across the genes in the cluster was compared with the average in other genotypes to using a Z-score measuring the difference from the average genotype for this cluster. The genotypes expression rank relative to all other genotypes is also reported (low rank corresponds to low expression). See materials and methods for more details. **Table S7**. Gene annotation information for genes connected to *su1* in the developmental network. Gene annotation information for direct neighbors of *su1* is listed, including GrameneID, classical maize names, locus and orthology information. **Table S8**. Gene annotation and KN1 Chip-Seq target status for genes in *kn1* subnetworks.
(ZIP)
