# Correction: Discovering Functional Modules across Diverse Maize Transcriptomes Using COB, the Co-Expression Browser

**DOI:** 10.1371/journal.pone.0120222

**Published:** 2015-03-13

**Authors:** The PLOS ONE Staff

The link and image for Fig. 4 are not correct. Please view the correct Fig. 4 here.

**Fig 4:**
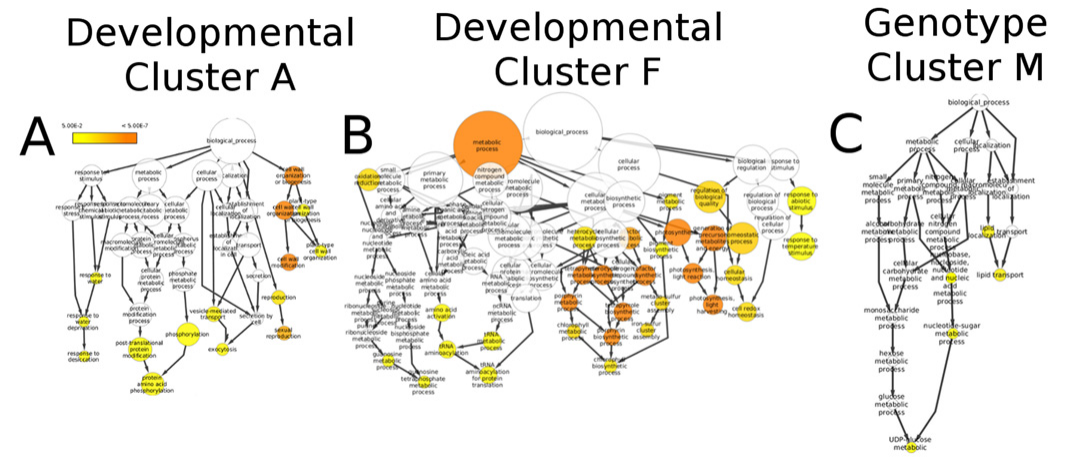
GO enrichment analysis of co-expression clusters. Gene clusters identified in Figure 2 were examined for enrichment of Gene Ontology terms. (A) Developmental cluster A, which exhibited a strong signal for expression in the anthers (see Figure 3), is enriched for GO terms related to sexual reproduction, response to desiccation, and cell wall biogenesis/modification. (B) Developmental cluster F, highlighted by patterns of expression in the leaves, is notably enriched for terms annotated for photosynthesis, response to temperature stimulus, and chlorophyll metabolism. (C) Genotype cluster M exhibits drastic under-expression in the P39 genotype, a sweet corn line, and shows significant GO enrichment in terms related to UDP-glucose as well as nucleotide-sugar metabolism and lipid transport

## References

[1] Schaefer RJ, Briskine R, Springer NM, Myers CL (2014) Discovering Functional Modules across Diverse Maize Transcriptomes Using COB, the Co-Expression Browser. PLoS ONE 9(6): e99193 doi: 10.1371/journal.pone.0099193 24922320 10.1371/journal.pone.0099193 PMC4055606

