# Supplementary figures and images for: Discovering Functional Modules across Diverse Maize Transcriptomes Using COB, the Co-Expression Browser
Source: PLoS One. 2014 Jun 12;9(6):e99193. doi: 10.1371/journal.pone.0099193 (PMC4055606; doi:10.1371/journal.pone.0099193)

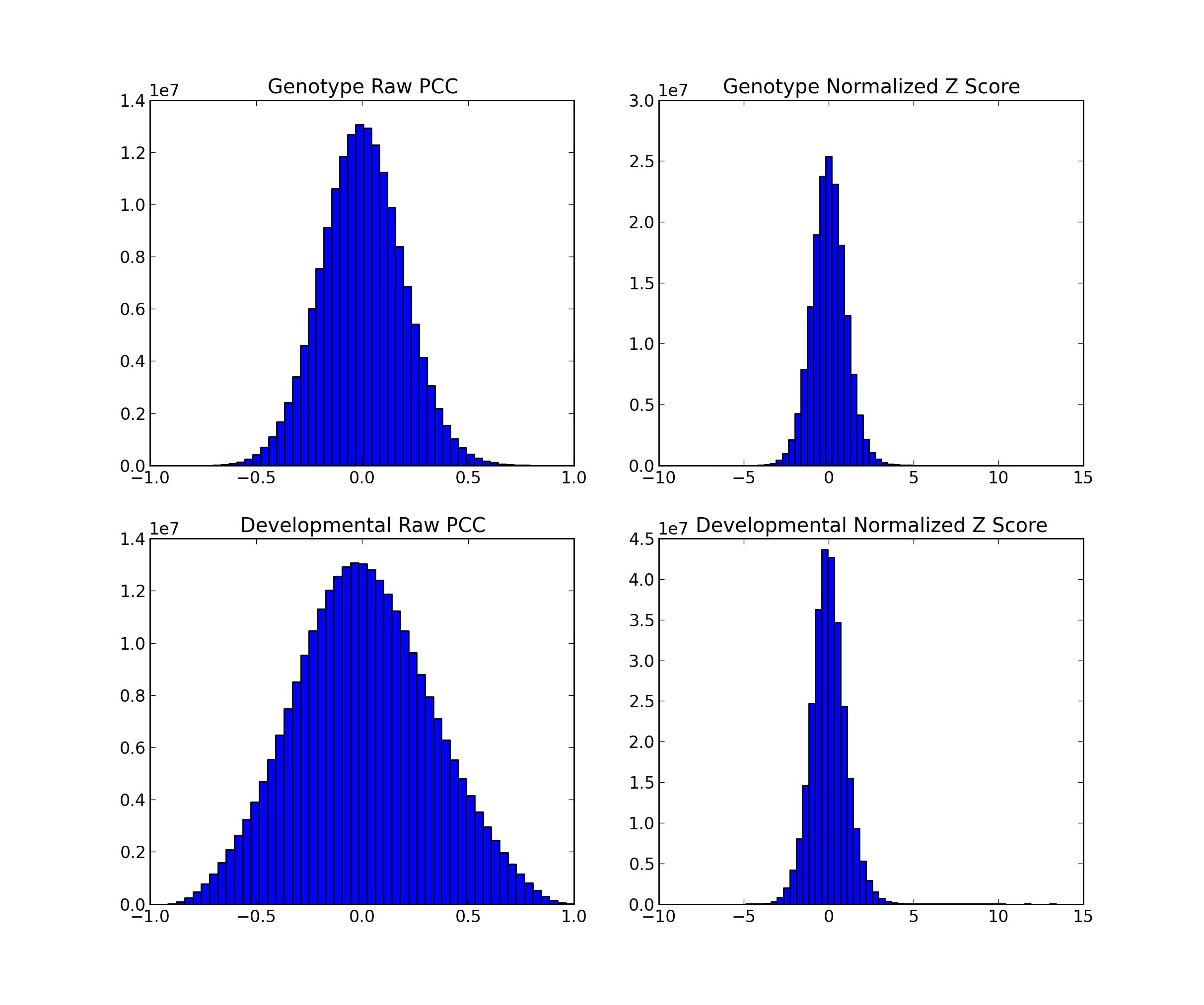

Supplement: Figure S1 — Normalization of pairwise correlation values. Histograms show pre- and post-normalized values for all pairwise interactions in genotype and developmental networks. Distributions are approximately normal, with the exception of heavy tails reflecting correlation structure among genes, and Z-score transformed distributions are comparable across networks. (TIFF) [file pone.0099193.s001.tiff]

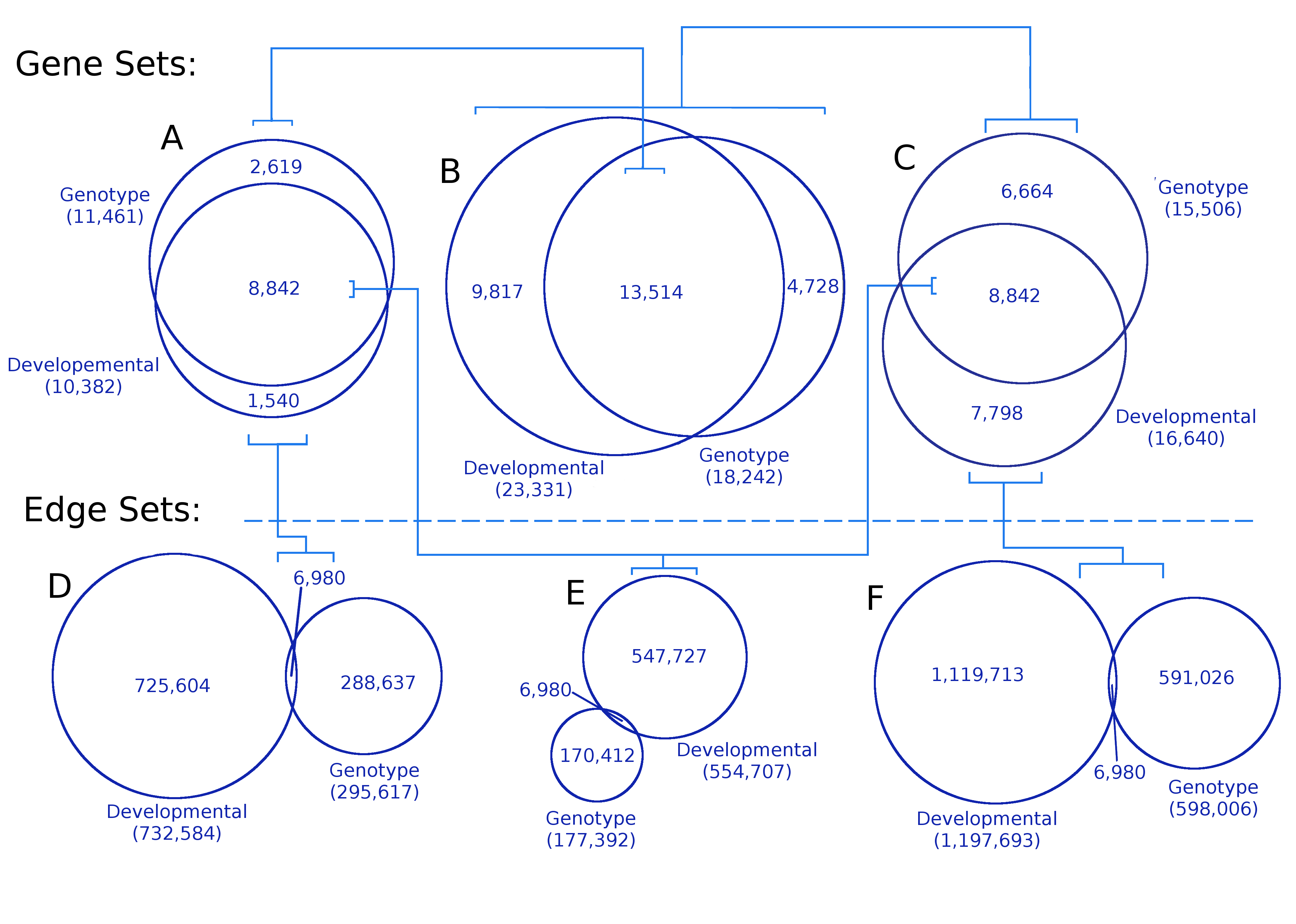

Supplement: Figure S2 — Comparison of gene sets in genotype and developmental network. Different starting sets of genes were used in the two experiments of which only 13,514 genes overlapped (A). Gene which retained at least a single significant co-expressed interaction are considered depending on whether the entire gene set was used (C) or only the union of the two data sets were considered (B). Similarly, corresponding edges were considered based on if they were calculated with all common genes (D), retained common genes (E), or simply all genes (F). (TIFF) [file pone.0099193.s002.tiff]

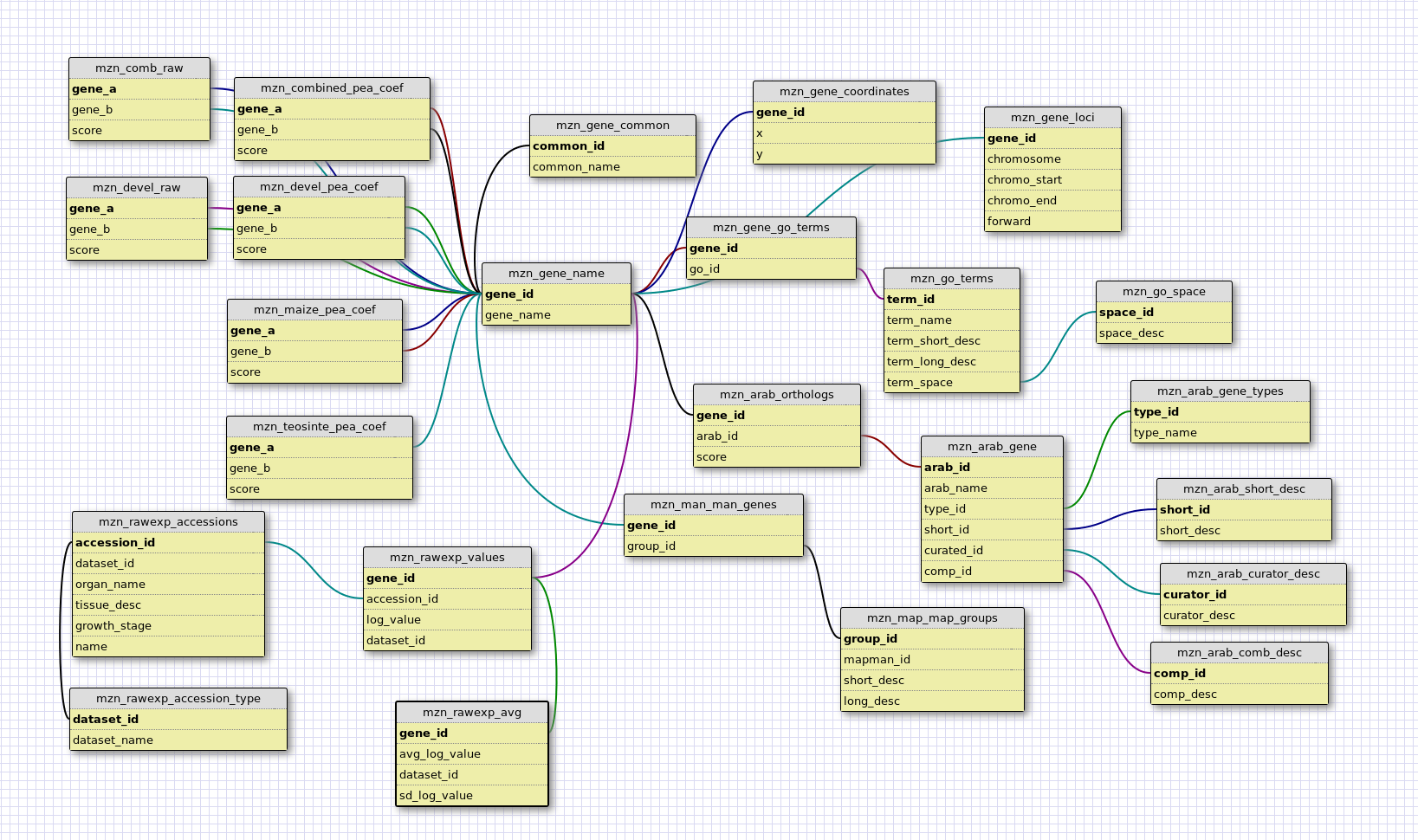

Supplement: Figure S3 — COB database schema. COB Database schema showing relationships among datatypes used in COB. Tables were designed both for query speed as well as optimized for large insertions. (TIFF) [file pone.0099193.s003.tiff]

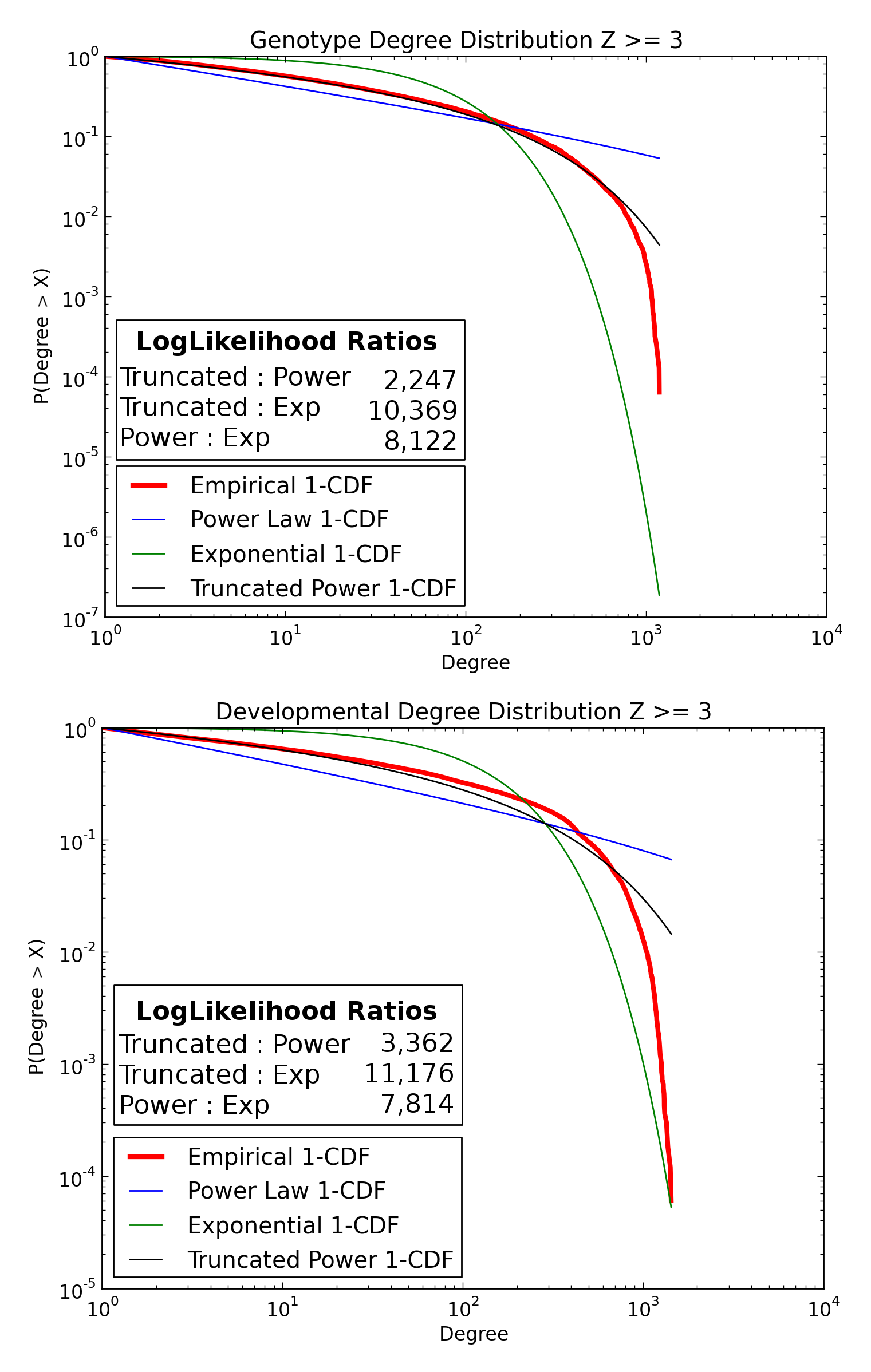

Supplement: Figure S4 — Network degree distributions and assessment of fit to candidate distributions. Degree distributions were assessed in each network individually at an edge significance cutoff of Z> = 3. Best fit lines for each distribution were plotted with degree against the probability of observing a degree larger than X. Bins were logarithmically spaced in order to control for the heavy tail. Loglikelihood ratios are inset comparing fits of different heavy tailed distributions commonly observed in other networks. Positive ratios reflect a higher likelihood of the first listed distribution. (TIFF) [file pone.0099193.s004.tiff]
